# Biosurfactant potential and antiviral activity of multistrain probiotics

**DOI:** 10.1016/j.heliyon.2023.e22837

**Published:** 2023-11-24

**Authors:** Tjie Kok, Denny Nyotohadi

**Affiliations:** Faculty of Biotechnology, University of Surabaya, Surabaya, 60293, Indonesia

**Keywords:** Biosurfactant activity, Antiviral activity, Multistrain probiotics, SARS-CoV-2, Virus infectivity

## Abstract

The COVID-19 caused by the SARS-CoV-2 has become a great threat to humans. However, there is no recommendation for an effective and safe drug to treat the disease. The strategy developed in this study is to utilize biosurfactant potential activity of *Lactobacillus* spp. and *Rhodopseudomonas palustris* probiotics to prevent the virus from entering human body. The outer membrane of the virus is comprising of phospholipid compounds. Biosurfactants, are known to have detergent-like properties (able to dissolve lipids) that are safe for *in vivo* use. Thus, the biosurfactant potential activity of the multistrain probiotics extract is expected to be able to disrupt the phospholipid membrane, resulting in the inactivity of the virus to infect human body. The biosurfactant potential activity of the probiotics extract was evaluated using oil spreading, drop collapse, and emulsification methods. The virus infectivity was evaluated on the SARS-CoV-2 of delta variant as a virus model. The results indicated that the probiotics extract has biosurfactant potential activity, able to inhibit virus growth up to 99.9 % within 48 h in the prevention platform, and up to 99.6 % within 48 h in the treatment platform. Therefore, the multistrain probiotics extract was identified to have potential as a promising antiviral agent.

## Graphical abstract

**Figure:**
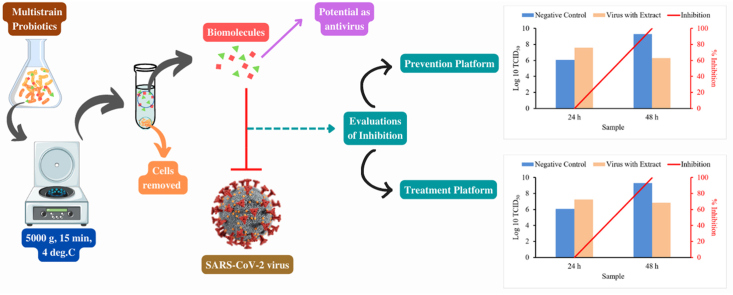

## 1 Introduction

COVID-19 is an infectious disease caused by the SARS-CoV-2. This outbreak began in 2019 in Wuhan city, China, and is suspected to have been transmitted by animals sold at the Huanan traditional market to human [1]. Transmission of the virus from human to human is through droplets released by sufferers when coughing, sneezing, or touching mouth, nose and eyes with hands. Many countries are trying to produce drugs to treat COVID-19. Not only synthetic drugs but also herbal medicines are being developed to handle the disease [2]. The demand for safe and effective drugs is an urgent need that must be met during the pandemic.

In October 2020, the Food and Drug Administration (FDA) granted an emergency use authorization to use remdesivir injection for COVID-19 treatment. Remdesivir exhibits antiviral activity by inhibiting virus replication. However, the medication with remdesivir is accompanied with harmful side effects for patients such as digestive, respiratory, cardiovascular, and kidney disorders [3,4]. In addition, the need for oral medications is a concern for practitioners. The use of oral medications will facilitate treatment and minimize the need for hospitalization. Many medications currently used to treat COVID-19, such as remdesivir, baricitinib, and corticosteroids, are administered as intravenous injections.

In October 2021, molnupiravir emerged as the first oral COVID-19 medication. The results of the third phase of clinical trials showed that molnupiravir was able to reduce the risk of death and hospitalization by up to 50 % [5]. Molnupiravir inhibits SARS-CoV-2 through mutations in the virus replication process. However, based on the study, it was stated that molnupiravir can cause mutations in mammalian cells, thus long-term use needs to be evaluated and monitored [6]. The World Health Organization (WHO) stated on its official website that there is no safe and effective drug for COVID-19 treatment (Off-label use of medicines for COVID-19, 2020). The uncertainties of dosage, efficacy, safety, and long-term effects make existing medications need to be further investigated before being used more extensively.

One of the approaches that could be taken to handle the problem is using biosurfactant potential activity of multistrain probiotics. Several microorganisms are known to produce compounds having biosurfactant (detergent-like properties) activity [7]. Such compounds can interact with phospholipid membrane and capsid of the virus. The formation of ion channels in the virus membrane can cause the loss of proteins involved in the process of infection and virus replication [8]. This interaction can also disrupt the membrane structure, leading to virus extermination [9]. Antiviral activity by biosurfactants will be even stronger if they have a fatty acid chain with 15 carbon atoms and 1 negative ion [10]. Study on SARS-CoV inactivation by heparin molecule showed that the virus envelope has a positive charge at the protein part of the membrane that can interact with negatively charged molecules, thus this interaction can prevent the virus from attaching to a target cell [11,12].

Biosurfactant compounds produced by *Lactobacillus plantarum* was reported to be able to inhibit the infection of the *Newcastle Disease Virus* (NDV) LaSota strain [13]. Extracts from *L.* *sakei* MN1 and *L*. *plantarum* LRCC5310 could kill *Infectious Pancreatic Necrosis Virus* (IPNV), *Infectious Hematopoietic Necrosis Virus* (IHNV) and *Human Rotavirus* strain WA [11]. Supernatant taken from *L*. *rhamnosus, L. acidophilus,* and *L. plantarum* could prevent the replication of several types of envelope viruses [14]. Biosurfactant surfactin from *Bacillus subtilis* could inhibit the replication of *Herpes Simplex Virus* (HSV) [15]. Sophorolipids from *Candida bombicola* as anti-HIV [16] and licenisin from *B*. *licheniformis* was reported as an anti-porcine epidemic diarrhea virus (PEDV) [17]. Clinical trials in several countries showed that biosurfactant compounds could be used as antiviral agent on *Acute Respiratory Distress Syndrome* (ARDS) [10].

Moreover, biosurfactants are reported as having acceptable properties for medication such as can be used orally and has a low cytotoxic level [8]. The results of a study using *L. jensenii* and *L. rhamnosus* extract showed that the biosurfactants in the extract has antimicrobial activities against *E. coli*, *A. baumannii*, and *S. aureus* at 25–50 mg/mL [18].

On the basis of the backgrounds, we conducted a research using extract solution of *Lactobacillus* spp. and *Rhodopseudomonas palustris* probiotics in developing antiviral medication. After being evaluated for its biosurfactant potential activity, the probiotics extract was further investigated for its potential to inactivate the virus. *In vivo* experiments are needed to assess the extract's safety and efficacy within living organisms before the probiotics being used more extensively.

## 2 Methods

### 2.1 Multistrain probiotics extract

AMRO Institute in Surabaya, Indonesia provided the multistrain probiotics which consist of *L. plantarum EMRO 009, L. casei EMRO 213, L. casei EMRO 002, L. rhamnosus EMRO 014, L. bulgaricus EMRO 212, L. fermentum EMRO 211,* and *R*. *palustris EMRO 201* in a fermented medium (honey of 1.13 % v/v, citric acid of 20 mg/mL, molasses of 3.07 % v/v and *Aloe vera* juice of 1.07 % v/v, and water). The pH at the beginning is 4 and at the end is 3.2. The fermentation is carried out without the existence of oxygen for ±45 days at 26 °C. The amount of individual microorganism existing in the formulation was ∼2 × 10^6^ CFU/mL. The extract solution was obtained by centrifugation (Sorvall Biofuge Stratos, Thermo Scientific, USA) at 4 °C, 5000 g, 15 min to separate it from pellet. The pellet was discarded and the extract solution was used for the assays.

### 2.2 Biosurfactant activity

The biosurfactant potential activity of the probiotics extract was evaluated using oil spreading, drop collapse, and emulsification methods.

In oil spreading method, a total of 50 mL of distilled water was poured into a Petri dish followed by the addition of 1 mL of olive oil on its surface. Next, 20 μL of the extract solution was dropped on the surface of the oil. The formation of a clear zone on the surface of oil indicates the biosurfactant potential activity of the extract solution.

In drop collapse method, 30 μL of the extract solution was dropped on the surface of the parafilm and left for 15 min. The droplet of extract solution that is getting bigger in diameter or becoming flat on the parafilm surface indicates the biosurfactant potential activity of the extract solution. Distilled water was used as a negative control and sodium dodecyl sulfate 1 % as a positive control.

In emulsification method, the extract solution was mixed with kerosene in a ratio of 1:1 (v/v) and vortexed for 2 min. The formation of emulsion indicates the biosurfactant potential activity of the extract solution. Distilled water was used as a negative control and sodium dodecyl sulfate 1 % as a positive control.

### 2.3 Cytotoxicity evaluation

Cytotoxicity of extract solution was evaluated on Vero E6 cells. Several concentrations (50 %, 33.3 %, 25 %, 20 %, and 16.6 % v/v) of extract solution were made by serial dilution of the extract solution with maintenance medium consisting of Dulbecco's Modified Eagle Medium (DMEM), Fetal Bovine Serum (FBS) 2 %, and penicillin-streptomycin 1 %. Then, 500 μL of trypsinated Vero E6 cells containing 1 × 10^6^ cells/well were put into each well of a 24 wells microplate. The cells were incubated at 37 °C, 5 % CO_2_ until they were confluent. After confluency, the medium was discarded, the cells were washed with PBS solution, and 1 mL of extract solution was put into each well. For negative control, phosphate buffer saline (PBS) solution was added to each well instead of the extract solution. After incubation for 72 h at 37 °C, 5 % CO_2_, the cells were fixed with 10 % of formalin for 30 min to prevent them from damage. The fixed cells were then stained with 2 % crystal violet and allowed to stand for 5 min. Further, the microplate was washed with running water, and gently tapped on a tissue paper until excess liquid was removed. After the microplate was dry, the dead cells appeared as transparent areas while living cells as purple stained areas.

### 2.4 Virus infectivity evaluation

Virus infectivity of the probiotics extract was evaluated to measure the extract activities to inhibit the virus growth, resulting in the reduction of infected Vero E6 cells. The virus infectivity evaluation of the probiotics extract was performed on SARS-CoV-2 of delta variant as a virus model using prevention and treatment platforms.

In the prevention platform, a 500 μL of Vero E6 cells containing 1 × 10^6^ cells/well in DMEM medium and 5 % FBS were put into 6 wells of microplate and incubated at 37 °C, 5 % CO_2_ until the cells were confluent. After confluency, the medium was discarded and the cells were washed with PBS solution. One mL of extract solution at a final concentration of 16.6 % v/v was then put into each well and the culture was incubated for 30 min. Subsequently, 200 μL of viruses with a TCID_50_ concentration of 1 × 10^6^ cells/mL was put into each well. Next, the cell mixture was incubated for 60 min at 37 °C, 5 % CO_2_. Afterwards, maintenance medium was put into each well and the cultures were incubated for 24 and 48 h. At 24 h and 48 h, 200 μL of the mixture was taken from each well for TCID_50_ evaluation. A negative control containing Vero E6 cells and PBS solution, and a virus control containing Vero E6 cells and viruses were also evaluated for TCID_50_ evaluation at the periods of incubation.

In the treatment platform, a 500 μL of Vero E6 cells containing 1 × 10^6^ cells/well in DMEM medium and 5 % FBS were put into 6 wells of microplate and incubated at 37 °C, 5 % CO_2_ until the cells were confluent. After confluency, the medium was discarded and the cells were washed with PBS solution. 200 μL of the SARS-CoV-2 with a TCID_50_ concentration of 1 × 10^6^ cells/mL was then put into each well. The culture was incubated for 60 min at 37 °C, 5 % CO_2_. Afterwards, 1 mL of extract solution with a final concentration of 16.6 % v/v was put into each well and the cultures were incubated for 24 and 48 h. At 24 h and 48 h, 200 μL of the mixture was taken from each well for TCID_50_ evaluation. A negative control containing Vero E6 cells and PBS solution, and a virus control containing Vero E6 cells and viruses were also evaluated for TCID_50_ evaluation at the periods of incubation.

### 2.5 TCID_50_ assay

Vero E6 cells containing 1 × 10^6^ cells/well were put in 48 wells microplate and incubated at 37 °C, 5 % CO_2_ until cells were confluent. After confluency, the medium was discarded and the cells were washed with PBS solution and ready for evaluation. The tested mixtures of the prevention and treatment platforms were diluted to give concentrations in the range of 1 × 10^−1^ to 1 × 10^−7^ using the maintenance medium. From each dilution, 100 μL of aliquot was taken and added into the prepared Vero E6 cells. The cultures were incubated for 60 min at 37 °C, 5 % CO_2_. Afterwards, the media was removed and the cells were washed with PBS solution. Next, maintenance media was added and the cultures were incubated at 37 °C, 5 % CO_2_ for 48–72 h until cytopathic effect appeared. At the established periods of 24 h, 48 h, or 72 h, the cells were fixed with 10 % of formalin and stained with 2 % of crystal violet. The value of TCID_50_ (cells/mL) virus titer was calculated using the Reed-Muench method [19].

**Figure:**
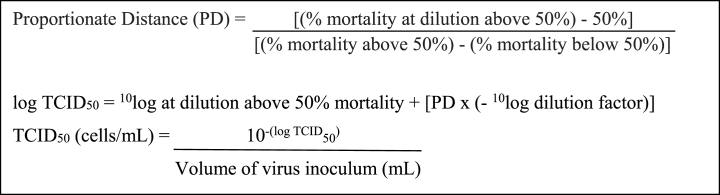

## 3 Results

### 3.1 Biosurfactant potential activity

#### 3.1.1 Oil spreading method

**Fig. 1:**
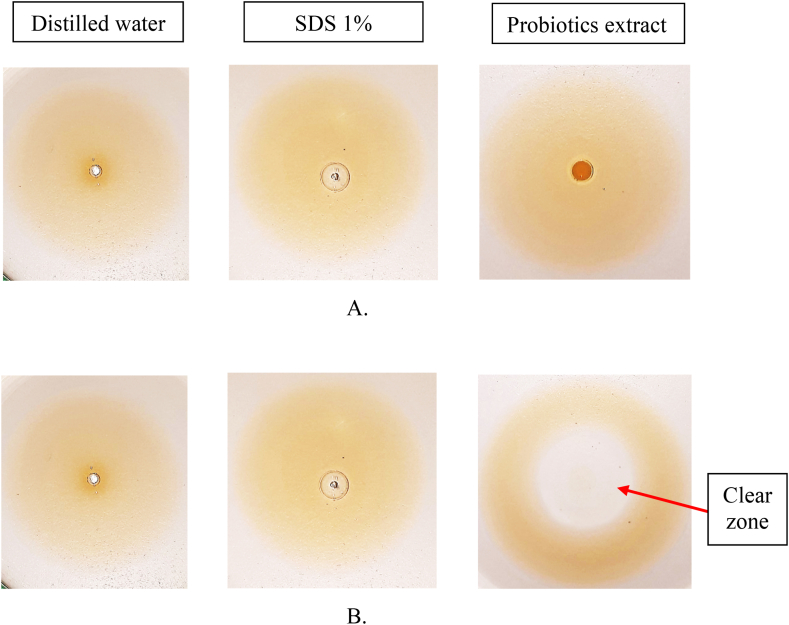
A. Appearance of the oil layer in the beginning during oil spreading method. B. Appearance of the oil layer after 15 min, clear zone indicating the biosurfactant potential activity of probiotics extract.

Distilled water, SDS 1 %, and probiotics extract was each dropped on top of the olive oil layer on Petri dish surface in the beginning (Fig. 1A). After 15 min (Fig. 1B), it was found that for probiotics extract the oil layer split to form a clear zone; the SDS 1 % (positive control), behaved likewise but the clear zone was formed directly soon after it was dropped. It indicated the biosurfactant potential activity of the probiotics extract, even though it is weaker than the SDS 1 % (positive control). The distilled water (negative control) gave no clear zone.

#### 3.1.2 Drop collapse method

**Fig. 2:**
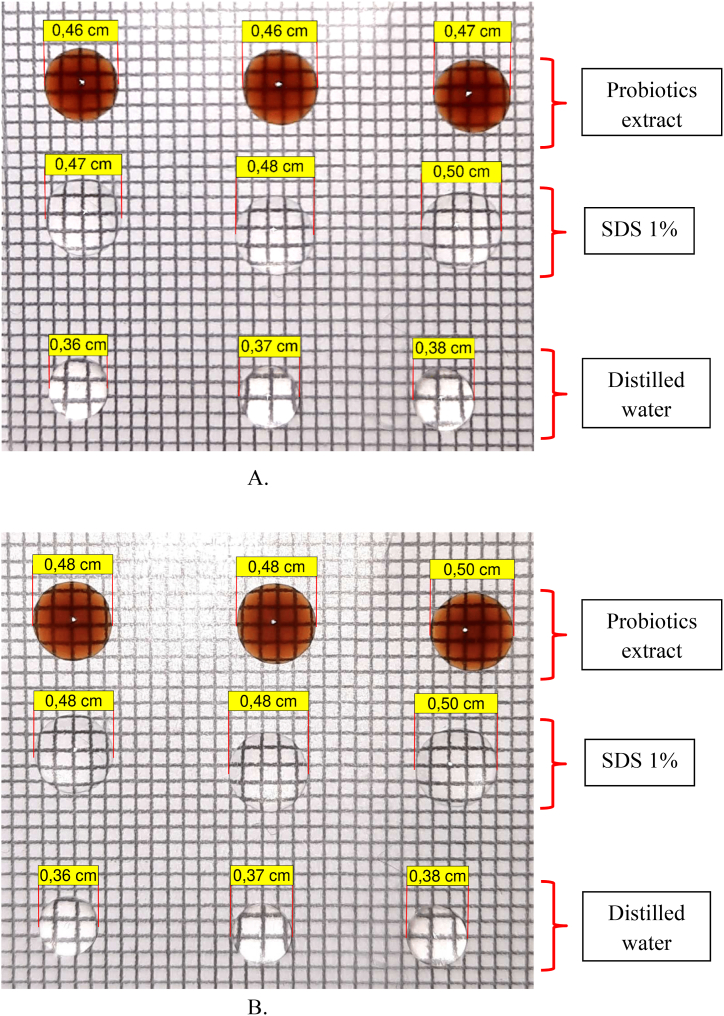
A. Droplet area in the beginning during drop collapse method. B. Droplet area after 15 min, broadening of droplet area indicating the biosurfactant potential activity of probiotics extract.

Distilled water, SDS 1 %, and probiotics extract was each dropped on the parafilm in the beginning (Fig. 2A). After 15 min (Fig. 2B), it was found that for probiotics extract the area of the droplet was broadened; for the SDS 1 % (positive control) the area of the droplet was also a bit broadened; and for distilled water (negative control) the area of the droplet remains unchanged. This indicated the biosurfactant potential activity of the probiotics extract.

#### 3.1.3 Emulsification method

**Fig. 3:**
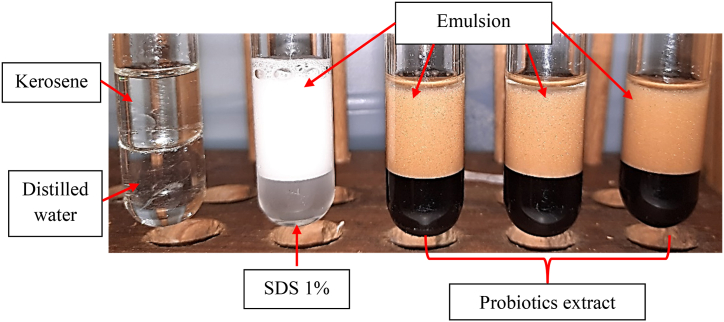
The emulsification (formation of emulsion), indicating the biosurfactant potential activity of test solutions.

Distilled water, SDS 1 %, and probiotics extract was each gently vortexed with kerosene (Fig. 3). It was found that for probiotics extract and SDS 1 % (positive control) the emulsion was formed, meanwhile for distilled water (negative control) no emulsion was formed. This indicated the biosurfactant potential activity of the probiotics extract.

#### 3.1.4 Cytotoxicity evaluation

**Fig. 4:**
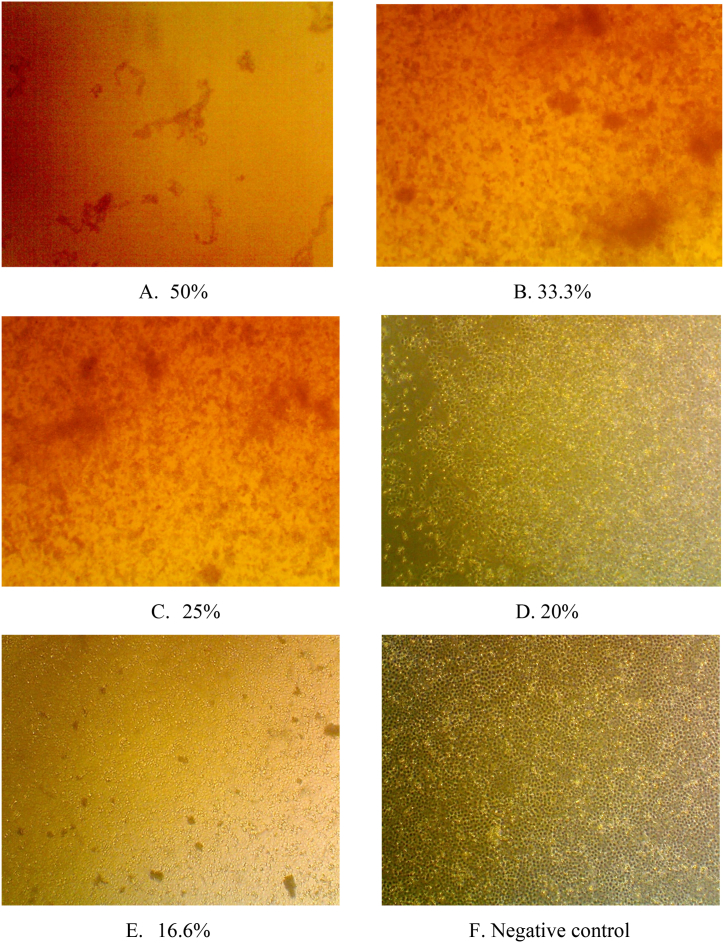
Observation of cytopathic effects using microscope with 10-fold magnification for probiotics extract at concentrations (v/v) of A. 50 %, B. 33.3 %, C. 25 %, D. 20 %, E. 16.6 %, and F. Negative control.

**Fig. 5:**
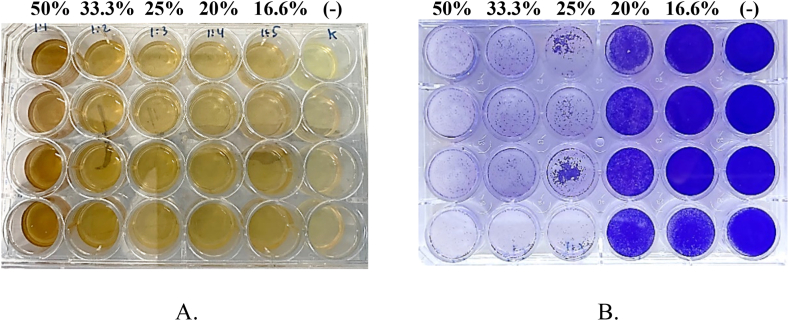
A. Before staining with crystal violet during cytotoxicity evaluation for probiotics extract at concentrations (v/v) of 50 %, 33.3 %, 25 %, 20 %, 16.6 %, and negative control (−). B. After staining with crystal violet, showing that the color intensity of the mixture of cells and the probiotics extract at the concentration of 16.6 % v/v is similar to that of the negative control.

The probiotics extract was diluted to obtain final concentrations (v/v) of 50 %, 33.3 %, 25 %, 20 %, and 16.6 %, and evaluated for their cytotoxicity on Vero E6 cells (Fig. 4A–F). No morphological changes (no cytopathic effects) was observed at the concentration of 16.6 % v/v (Fig. 4E) similar to that of the negative control (Fig. 4F). The crystal violet staining results (Fig. 5A and B) showed that the color intensity of the mixture of cells and the probiotics extract at the concentration of 16.6 % v/v is similar to that of the negative control (Fig. 5B). This indicated that the probiotics extract at that concentration is non-toxic to the cells.

#### 3.1.5 Virus infectivity evaluation, prevention platform

**Fig. 6:**
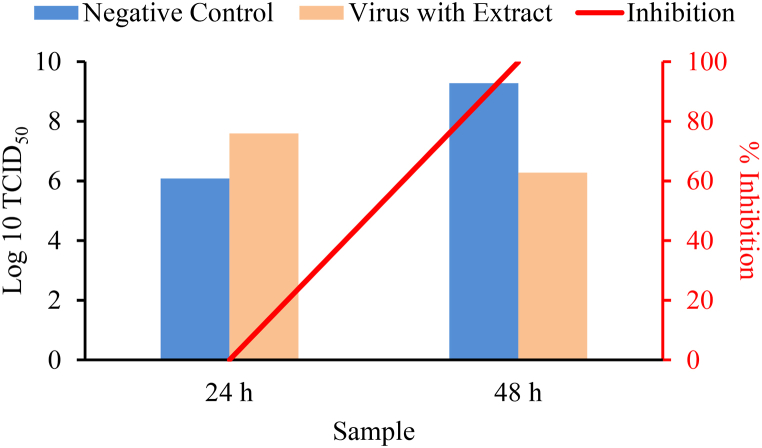
Log10 TCID50 and % Inhibition by probiotics extract in the prevention platform.

The Vero E6 cells were first incubated with probiotics extract, the viruses was added afterwards, and the cultures were incubated further. At 24 h and 48 h, the probiotics extract was found to give an average TCID_50_ of 3.9 × 10^7^ cells/mL and 1.9 × 10^6^ cells/mL, respectively, and at 48 h it was found to inhibit the growth of virus up to 99.9 % (Table 1, Fig. 6).

**Table 1:** TCID_50_ value and % Inhibition by probiotics extract in the prevention platform.

Sample	TCID_50_ (cells/mL)	Average TCID_50_ (cells/mL) ± SD	% Inhibition
Negative control 24 h	2.0 × 10^6^	1.2 × 10^6^ ± 0.8 × 10^6^	**-**
	3.7 × 10^5^		
	1.2 × 10^6^		
Virus with extract 24 h	3.9 × 10^7^	3.9 × 10^7^ ± 1.1 × 10^7^	0
	5.0 × 10^7^		
	2.8 × 10^7^		
Negative control 48 h	1.8 × 10^8^	1.9 × 10^9^ ± 1.8 × 10^9^	–
	3.7 × 10^9^		
	1.9 × 10^9^		
Virus with extract 48 h	3.7 × 10^6^	1.9 × 10^6^ ± 1.8 × 10^6^	99.9 %
	1.8 × 10^5^		
	1.9 × 10^6^

#### 3.1.6 Virus infectivity evaluation, treatment platform

**Fig. 7:**
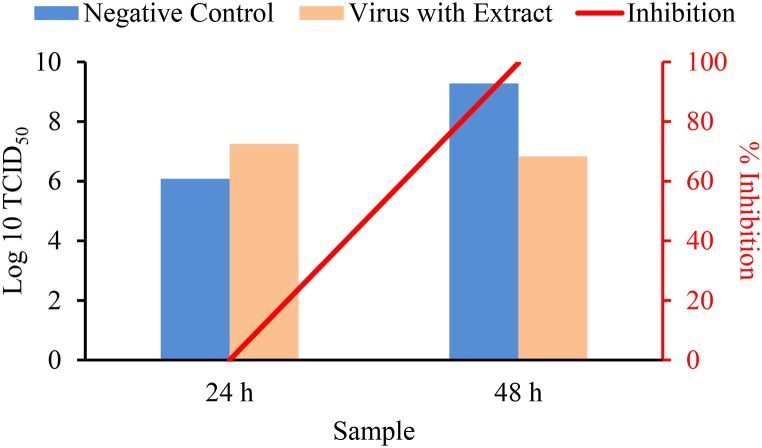
Log10 TCID_50_ and % Inhibition by probiotics extract in the treatment platform.

The Vero E6 cells were incubated with the viruses, the extract solution was added afterwards, and the cultures were incubated further. At 24 h and 48 h, the probiotics extract was found to give an average TCID_50_ of 1.8 × 10^7^ cells/mL and 4.8 × 10^6^ cells/mL, respectively, and able to inhibit the growth of virus up to 99.6 % (Table 2, Fig. 7).

**Table 2:** TCID_50_ value and % Inhibition by probiotics extract in the treatment platform.

Sample	TCID_50_ (cells/mL)	Average TCID_50_ ± SD (cells/mL)	% Inhibition
Negative control 24 h	2.0 × 10^6^	1.2 × 10^6^ ± 0.8 × 10^6^	**-**
	3.7 × 10^5^		
	1.2 × 10^6^		
Virus with extract 24 h	1.8 × 10^7^	1.8 × 10^7^ ± 0.0	0
	1.8 × 10^7^		
	1.8 × 10^7^		
Negative control 48 h	1.8 × 10^8^	1.9 × 10^9^ ± 1.8 × 10^9^	–
	3.7 × 10^9^		
	1.9 × 10^9^		
Virus with extract 48 h	5.0 × 10^6^	4.8 × 10^6^ ± 4.0 × 10^6^	99.6
	6.8 × 10^5^		
	8.7 × 10^6^

## 4 Discussion

The ability of probiotics to regulate body immune responses has made probiotics widely applied to treat diseases. In addition, the biosurfactant activity given by probiotics metabolites is also promising to explore in order to inhibit pathogen invasion. Up to date, it is only a small fraction that has been explored regarding the way by which the probiotics do their actions. In this study, the multistrain probiotics containing a mixture of lactic acid bacteria (*L. plantarum, L. casei, L. rhamnosus, L. bulgaricus, L. fermentum*) and *R. palustris* were evaluated for the ability to inactivate viruses.

Lactic acid bacteria themselves are rod-shaped Gram-positive bacteria whose metabolites have good effects on human, including for antimicrobial applications. *R. palustris* belonging to the Gram-negative bacteria is known as a species which capable of catabolizing various energy sources in various conditions and producing high-quality products [20]. The symbiosis between this bacteria and lactic acid bacteria can increase the growth of lactic acid bacteria and increase the production of metabolites in the fermentation process [21]. Among the metabolites that play a role for pathogen inactivity are biosurfactants. With the biosurfactant activity, the metabolites is able to interact with the lipid membranes of pathogens, e.g. viruses, thus the probiotics has the potential to be developed as antiviral agents with a low level of cytotoxicity [16]. Therefore, the aim of the work was to evaluate the biosurfactant potential activity of multistrain probiotics extract and investigate its potential to inactivate viruses.

The probiotics extract was obtained by centrifugation to separate it from the pellet. The upper phase, i.e. the probiotics extract, was evaluated for its biosurfactant potential activity using oil spreading, drop collapse and emulsification methods. In the oil spreading method, the probiotics extract was dropped on top of the olive oil layer (Fig. 1A). After 15 min (Fig. 1B), the oil layer split to form a clear zone which indicates the biosurfactant potential activity that make the oil and water miscible and hence forms a clear zone [22]. This method is one of the best methods that can be used to detect the biosurfactant activity [23]. In the drop collapse method, the probiotics extract was dropped on the parafilm (Fig. 2A) and left for 15 min (Fig. 2B). The results showed that the droplet of the extract was broadened or flattened. The broadening or flattening of the droplet indicated the biosurfactant potential activity, which is able to decrease the surface tension between water and hydrophobic surface of the parafilm [22,24]. In the emulsification (formation of emulsion) method, the probiotics extract was vortexed with kerosene. The results showed that the emulsion is formed between the probiotics extract and kerosene(Fig. 3). Emulsification is a process in which two immiscible liquids form a miscible mixture when combined each other [23]. This process is enabled by the biosurfactant activity of the probiotics extract which is able to reduce the surface tension between the two immiscible liquids [22].

After being evaluated for its biosurfactant potential activity, the probiotics extract was tested for its cytotoxicity on Vero E6 cells. The extract solution was diluted using a maintenance medium to give final concentrations (v/v) of 50 %, 33.3 %, 25 %, 20 %, and 16.6 %, and tested for their cytotoxicity on Vero E6 cells. Microscopic observation (Fig. 4) showed that at concentrations of 50 %–25 % the extract was toxic to cells, as shown by cell shrinkage which results in inability of cell to attach and hence washed off during staining process. While at concentration of 20 % and 16.6 % v/v, the cell remained in good structure and attached in microplate base. The staining with crystal violet (Fig. 5B) showed that at concentrations of 50 %–25 % the extract was toxic to cells, as shown by colorless or unstained areas on the microplate, which indicates that during incubation with the extract, many cells were dead and washed off during staining. At concentration of 20 %, the level of extract toxicity decreased, as shown by faint violet color and at concentration of 16.6 % v/v the extract was considered as non-toxic which is shown by the violet color intensity that is similar to the negative control.

The potential of probiotics extract to inactivate virus was evaluated by infectivity test on SARS-CoV-2 of delta variant. In this evaluation, Vero E6 cells were used as host cells for the virus growth. Probiotics extract with a concentration of 16.6 % v/v, which has been shown to be non-toxic to cells, was used as the test solution. The results indicated that the probiotics extract was able to inhibit the growth of the virus.

In the prevention platform, the virus titer as an important tool for evaluation of virus infectivity by probiotics extract was determined after the Vero E6 cells were first incubated with the extracts, then the viruses were added and the cultures were incubated further. The incubation period for 24 h gave an average TCID_50_ of 3.9 × 10^7^ cells/mL with 0 % of virus inhibition and that for 48 h gave an average TCID_50_ of 1.9 × 10^6^ cells/mL with 99.9 % of virus inhibition, compared to that of the negative control of 1.9 × 10^9^ cells/mL (Table 1, Fig. 6). The virus inhibition was calculated based on the formula:

$$\%\;inhibition=\frac{negative\;control\;TCID50-\;mixture\;with\;extract\;TCID50}{negative\;control\;TCID50}\times 100\%$$

In the treatment platform, the virus infectivity by probiotics extract was determined after the Vero E6 cells were first incubated with the viruses, and then the extracts were added and the cultures were incubated further. The incubation period for 24 h gave an average TCID_50_ of 1.8 × 10^7^ cells/mL with 0 % of virus inhibition and that for 48 h gave an average TCID_50_ of 4.8 × 10^6^ cells/mL with 99.6 % of virus inhibition, compared to that of negative control of 1.9 × 10^9^ cells/mL (Table 2, Fig. 7).

For the prevention platform, the results showing the average TCID_50_ for 24 h of 3.9 × 10^7^ cells/mL with 0 % of virus inhibition indicated that in the period of time the viruses are not inhibited, but growing even slightly faster than that of the negative control (with the average TCID_50_ of 1.2 × 10^6^ cells/mL). Meanwhile the average TCID_50_ for 48 h of 1.9 × 10^6^ cells/mL indicated that the growth of viruses is inhibited. The calculated virus inhibition by the extract reached 99.9 %.

Likewise, for treatment platform the results showing the average TCID_50_ for 24 h of 1.8 × 10^7^ cells/mL with 0 % of virus inhibition indicated that in the period of time the viruses are not inhibited, but growing even slightly faster than that of the negative control (with the average TCID_50_ of 1.2 × 10^6^ cells/mL). Meanwhile the average TCID_50_ for 48 h of 4.8 × 10^6^ cells/mL indicated that the growth of viruses is inhibited. The calculated virus inhibition by the extract reached 99.6 %.

Taken together, the multistrain probiotics (*Lactobacillus* spp. and *R. palustris*) extract showed antiviral activity against the SARS-CoV-2 of delta variant, inhibiting the virus by 99.9 % in the prevention platform and 99.6 % in the treatment platform within 48 h. Clinical trials and long-term studies are required to evaluate the extract's safety, effectiveness, and resistance development in treating COVID-19 in humans.

Given the average incubation time for the SARS-CoV-2 in the human body of 2–11 days (De Vito et al., 2022), the probiotics extract could be regarded as promising to be used for virus inactivation thus prevent the body from the virus infection and from the damages arising therefrom. The virus inactivity could probably be exerted by biosurfactant activity of the extract's metabolites. Detailed investigations are required to fully understand how the biosurfactants interact with and inhibit the virus.

## 5 Conclusion

The extract of *Lactobacillus* spp. and *Rhodopseudomonas palustris* probiotics showed biosurfactant potential activity which was identified by oil spreading, drop collapse, and emulsification methods. The virus infectivity evaluation showed that in the prevention and treatment platforms the extract with a concentration of 16.6 % v/v is able to give an inhibition on the growth of the SARS-CoV-2 of delta variant within 48 h with TCID_50_ of 1.9 × 10^6^ cells/mL and 4.8 × 10^6^ cells/mL, and % virus inhibition of 99.9 and 99.6 %, respectively. The virus inactivity could probably be exerted by the biosurfactant activity of the extract. Hence, the multistrain probiotics extract has potential for an antiviral agent.

## Funding

No funding was received for the work.

## Data availability statement

Data associated with this study has not been deposited into a publicly available repository. Data will be made available on request.

## Additional information

No additional information is available for this paper.

## CRediT authorship contribution statement

**Tjie Kok:** Conceptualization, Data curation, Formal analysis, Funding acquisition, Methodology, Resources, Supervision, Writing - original draft, Writing - review & editing. **Denny Nyotohadi:** Formal analysis, Investigation, Visualization.

## Declaration of competing interest

The authors declare that they have no known competing financial interests or personal relationships that could have appeared to influence the work reported in this paper.

## List of abbreviations

TCID50: Tissue Culture Infectious Dose 50
